# The neurocognitive and BDNF changes of multicomponent exercise for community-dwelling older adults with mild cognitive impairment or dementia: a systematic review and meta-analysis

**DOI:** 10.18632/aging.102918

**Published:** 2020-03-19

**Authors:** Xinyi Wang, Haiyun Wang, Zhenghui Ye, Guofei Ding, Fengli Li, Ji Ma, Wei Hua

**Affiliations:** 1 Department of Anesthesiology, Tianjin Third Central Hospital, Nankai University Affinity the Third Central Hospital, The Third Central Clinical College of Tianjin Medical University, Tianjin Institute of Hepatobiliary Disease, Tianjin Key Laboratory of Extracorporeal Life Support for Critical Diseases, Tianjin 300170, China

**Keywords:** aged, multicomponent exercise, community dwellers, group exercise, cognitive impairment

## Abstract

Our goal was to examine whether multicomponent exercise performed by older adults with mild cognitive impairment or dementia as group-based exercise in community have beneficial effects on cognition and brain-derived neurotrophic factor. Eight studies were identified through Emabase, Medline, PubMed. Searches combined terms for neurocognitive and biochemical changes with those for MCI and dementia. Data were extracted and checked by a second reviewer, systematically reviewed, and meta analyzed where appropriate. There was significant difference in favor of multicomponent exercise in cognition (WMD:0.18; 95%CI:0.02-0.34), attention (SMD=2.16; 95%CI:1.2to3.12) and executive function (SMD =0.80; 95%CI: 0.28to1.31), but not in memory. However, there was limited reporting of the effects of multicomponent exercise on depression and brain-derived neurotrophic factor for this group of people. In conclusion, this meta-analysis indicated that group exercises improve cognition, attention and executive function in community-dwelling older adults with mild cognitive impairment or Alzheimer's disease.

## INTRODUCTION

There are 47.5 million people with dementia worldwide, and the number is expected to rise to 131.5 million by 2050 [1]. Older adults with mild cognitive impairment are more likely to experience worsening of functional agility, cognitive function and social involvement with aging, the incidence of MCI progression to dementia is 10 times that of the same age group [2–4]. Therefore, it’s important to ameliorate cognitive decline and impairment. Individuals who carry out an exercise program compared with sedentary ones have more advantage in mood improvement, cognition, brain plasticity, neurotransmitters’ production [5, 6]. Long-term benefit of nonpharmacologic therapy has been more and more apparent, and could be a very valuable choice for delaying the progression of the disease on global cognitive functions in patients with AD (Alzheimer's disease) [7]. In China, the prevalence rate of dementia is 5.4% for people aged over 60 years. More than 80% suffer from mild dementia among older adults with dementia and live in the community, while only 11% have been diagnosed with dementia [8, 9]. Previous studies demonstrate that reduced social participation has been relevant to cognitive deficit and higher risk of dementia, however, group exercises in possible patterns such as exercise programs closer to home can keep old people functionally active and independence and enable them to get more social interaction, make people in this age bracket more practical to participate in and increase adherence and acceptance to the programs [10–12]. Encouraging older adults to take part in community activities is the countermeasure put forward in many countries to face aging of population and cutting down public health costs, which is particularly related to the local destitute and socially vulnerable population [13–15]. Considering the growing number of published studies newly and the increasing interests concerning this issue, the aim of this systematic review with meta-analysis was to investigate the effects of community-based multicomponent exercise(ME) on neurocognitive and neurotrophic biomarkers of community dwellers with MCI or dementia.

## RESULTS

### Characteristics of included studies

**Figure 1:**
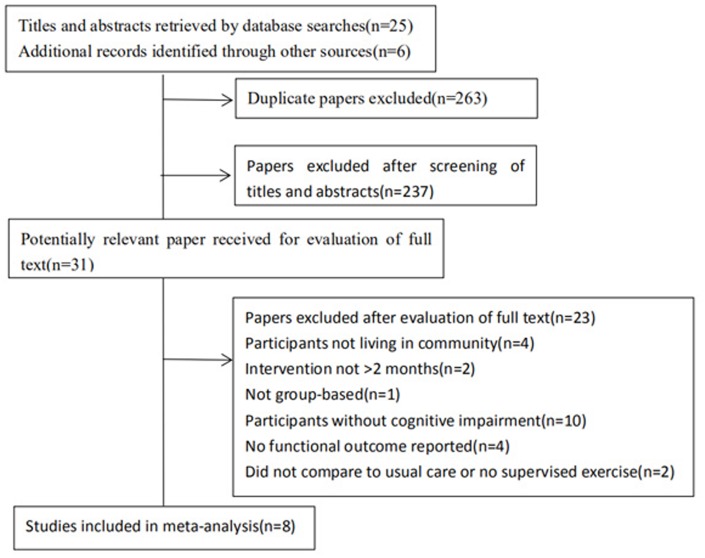
**Flow diagram of the trial identification process.**

Initial database searching and reference list scanning identified 531 studies, with 268 remaining after the removal of duplicates (Figure 1). Inclusion and exclusion criteria were applied to the full texts, finally, seven randomized controlled trials and one prospective cohort study were included in this meta-analysis (Table 1) [16–23]. There were 636 participants included of this meta, the mean age of participants ranged from 68 to 81 years. The mean baseline Mini-Mental State Examination score among participants ranged from 12.4 to 26.7 (only one trail used MoCA score at 21.8). The intervention duration was around two months in two trials, six months in four trials and twelve months in two trials. All 8 trials included multicomponent exercise as the intervention. The content of the exercise programs was multicomponent (with a combination of stretching, strengthening, aerobic, balance, Tai chi and mental or cognitive exercises. In one trial, participants performed a biking program with visual reality). Considering the intensity of aerobic exercise, moderate to vigorous intensity was used in most of the studies analyzed. Control of the intensity of intervention programs were based on maximum heart rate around75%. The frequency of exercise of included studies varied from one to four sessions a week. Exercise sessions were between 0.5 and 4 hours long and included warm ups and cool downs. The programs were supervised by physiotherapists with experience in geriatric rehabilitation or dementia, or by trained carers, coaches or stuff members. Comparators included: normal care [16–18], education or interest classes [19–23], and only one was video game [23]. There was a limited variety of biomarkers assessed within studies. Considering neurotrophic factors, BDNF (brain-derived neurotrophic factor) was the outcome of three of eight selected studies [17, 22, 23]. Drop-out rates of participants receiving an exercise intervention were reported in all trials and ranged from 6% to 33%, with a combined drop-out rate of 12%. Participant adherence rates ranging between 75% and 99%.

**Table 1:** Modified Jadad score.

**Study**	**Randomization**	**Allocation concealment**	**Blinding**	**Withdraw**	**Score**
Hannareeta 2016 ^14^	2	2	1	1	6
Arsalan 2017 ^15^	2	2	1	1	6
Chih 2017 ^16^	0	2	0	1	3
Takao 2012 ^18^	2	2	1	1	6
Seongryu 2019 ^19^	2	2	1	1	6
Daniel 2018 ^20^	2	2	1	1	6
Takao 2013 ^21^	2	2	1	1	6
Cay 2018 ^22^	2	1	0	1	4

### Assessment of risk of bias

The included trials had a mean Jadad score of 5 (Table 2). As these trials were unlikely to meet blinding requirements of therapists and patients, a maximum score of 7 was expected. The Grades of Recommendation, Assessment, Development and Evaluation (GRADE) approach was applied to evaluate the quality of the body of evidence in each meta-analysis (Table 3). The quality of each body of evidence was downgraded or upgraded from the baseline high quality according to a set of predefined criteria. The levels of quality were very low, low, moderate, or high quality. Evidence was downgraded two places if most trials scored 4, indicating poor methodological quality. Evidence quality was upgraded one place if the effect size was large. For indirectness, as indicated by varied participant populations or interventions; for inconsistency, as indicated by an I^2^ value > 50% that could not be explained in sensitivity analyses, indicating substantial heterogeneity, or for imprecision of results, as indicated by a wide 95% CI.

**Table 2:** GRADE score.

**Outcome**	**Design**	**Risk of bias**	**Indirectness**	**Inconsistency**	**Imprecision**	**Publication Bias**	**Effect size**	**GRADE quality**
Cognition	RCT*5	-1	0	0	0	-1	0	++
Memory	RCT*6	0	0	-1	-1	0	0	++
Attention	RCT*4	-1	0	0	0	0	0	+++
Executive function	RCT*4	-1	0	0	0	0	+1	+++

**Table 3:** Study characteristics of the included studies.

**Study**	**Country**	**Participants**	**Intervention**	**Outcome measures**
Hannareeta 2016 [16]	Finland	N=140 Age=78 Gender=89M, 51F MMSE=18.1 Diagnose=AD	Exp= multicomponent exercise, 4h * 2d / wk * 12mth,Community setting; Group; Supervised by physiotherapists. Con=usual care; oral and written advice on nutrition and exercise methods.	MMSE; CVFT; CDR; CDT. Follow up=0,3,6mon.
Arsalan 2017 [17]	Iran	N=22 Age=68 Gender=0M, 24F MMSE=24 Diagnose=MCI	Exp= multicomponent exercise, 60min * 3d / wk * 8weeks(total of 24 sessions), Community setting; Group; Supervised by stuff members. Con=usual care.	GDS, DST, DSC, SCWT, BDNF. Follow up=0,6mon.
Chih 2017 [18]	China	N=147 Age=81 MMSE=12.4 Diagnose=AD	Exp= multicomponent intervention, 2times/week*12 mth,Community setting; Group; Supervised by physiotherapists. Con=usual care.	MMSE, GDS, CDR; ADL. Follow up=0,12mon.
Takao 2012 [19]	Japan	N=50 Age=74 Gender=27M, 23F MMSE=26.7 Diagnose=MCI	Exp= multicomponent exercise, 90min *2d / wk * 12mth (total of 80 times), Community setting; Group; Supervised by physiotherapists and instructors. Con=Three education classes regarding health promotion during the 12-month study period, but no information regarding exercise, physical activity, or cognitive health.	MMSE; WMS-LM; DSC;VFT; SCWT. Follow up=0,6,12mon.
Seongryu 2019 [20]	Japan	N=83 Age=77 Gender=43M, 40F MMSE=26.7 Diagnose=MCI	Exp= multicomponent exercise, 90min * 2d / wk * 24 wk(total of 48 times), Community setting; Group; Supervised by stuff members. Con=Two health education classes (90 min * 24 week) about oral care and nutrition by a professional lecturer while sitting, no specific information regarding physical, cognitive, and social activities.	MMSE; CWM; SWM; TMT; SDST; GDS. Follow up=0,24wk.
Daniel 2018 [21]	China	N=80 Age=80 Gender=15M, 65F MMSE=20.4 Diagnose=Probable dementia	Exp= multicomponent exercise, 80min * 2d / wk(total of 14 sessions), Community setting; Group; Supervised by experts. Con=TAU provided by the elderly centers (interest classes and recreational activities), but excluding cognitive training and tai chi.	MMSE, DRS. Follow up=0,2mon.
Takao 2013 [22]	Japan	N=100 Age=75 Gender=51M, 49F MMSE=26.55 Diagnose=MCI	Exp= multicomponent exercise, 90min * 2d / wk * 6 month, Community setting; Group; Supervised by physiotherapists and instructors. Con=Two education classes regarding health promotion during the 6-month study period, but no information regarding exercise, physical activity, or cognitive health.	MMSE; ADAS; WMS-LM; BDNF. Follow up=0,6mon.
Cay 2018 [23]	Greece	N=14 Age=78 Gender=7M, 7F MoCA=21.8 Diagnose=MCI	Exp= multicomponent exercise, 45min * 4d / wk * 6mth, Community setting; Group; Supervised by stuff members. Con=game-only.	MoCA; TMT; SCWT; DST; ADAS; BDNF. Follow up=0,3,6mon.

ADAS: Alzheimer’s Disease Assessment Scale; CDR: Clinical Dementia Rating; CDT: Clock Drawing Test; CWM: Composite Word Memory; DRS: Dementia Rating Scale; DSC: Digit Symbol Coding; DST: Digit Span Test; GDS: Geriatric Depression Scale-15; L/CVF: Letter/Categorical Verbal Fluency; MMSE: Mini-Mental State Examination; MoCA: Montreal Cognitive Assessment; SCWT: Stroop Color Word test; SDST: Symbol Digit Substitution Test; SWM: Spatial Working Memory; TMT: Trail Making Test; VFT: Verbal Fluency Test; WMS-LM: Logical Memory Subtest of the Wechsler memory scale-revised.

### Outcomes

### Global cognitive function

**Figure 2:**
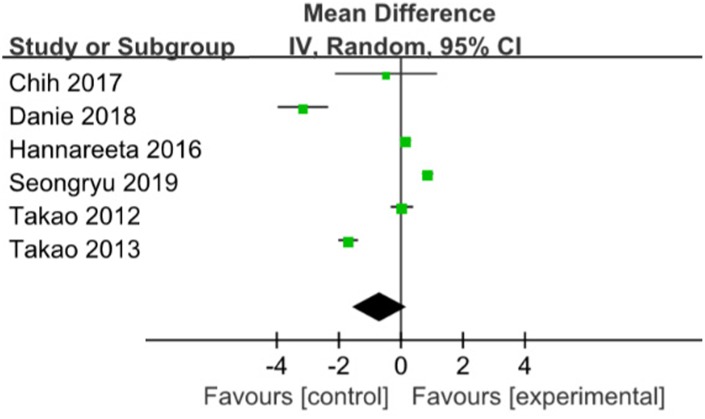
**The assessment of the effects of multicomponent exercise on global cognitive function by meta-analysis.**

Six trials with 546 patients investigated the effects of multicomponent intervention on global cognitive function using MMSE (Mini-Mental State Examination) scores. Global cognitive function was no significantly improved in the intervention groups (WMD= -0.68; 95%CI: -1.58 to 0.22), with high heterogeneity existed (P<0.01, I2= 98%) [16, 18–22] (Figure 2). However, three studies with multicomponent training duration of 12 months [16, 18, 19] were included for subgroup analysis (WMD:0.18; 95%CI: 0.02 to 0.34; p:0.03), with heterogeneity of 0% (p= 0.65), this result is in favour of the positive effect of multicomponent intervention on global cognitive function.

### Memory

**Figure 3:**
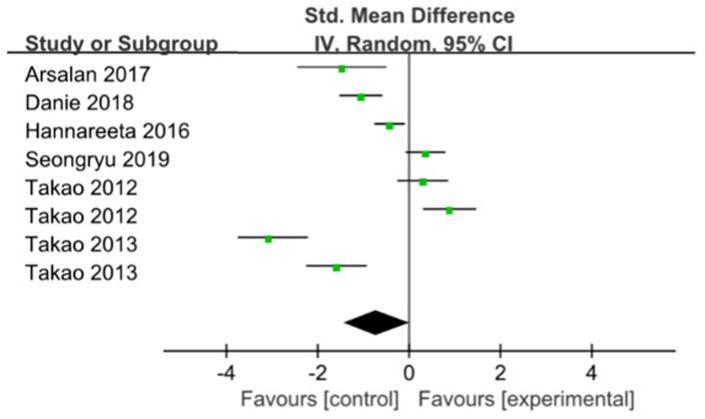
**The assessment of the effects of multicomponent exercise on memory by meta-analysis.**

Six studies were pooled with no significant effect in favor of ME for improving memory [16, 17, 19–22] (SMD= -0.71; 95% CI:-1.43 to 0.01; I2=93%) (Figure 3). When the trial with the smallest sample size and highest risk of bias [17, 22] was removed in a sensitivity analysis, a stable result was found between group compared with the control group (SMD=-0.01; 95% CI:-0.63 to 0.62), with stable heterogeneity of 89%.

### Attention

**Figure 4:**
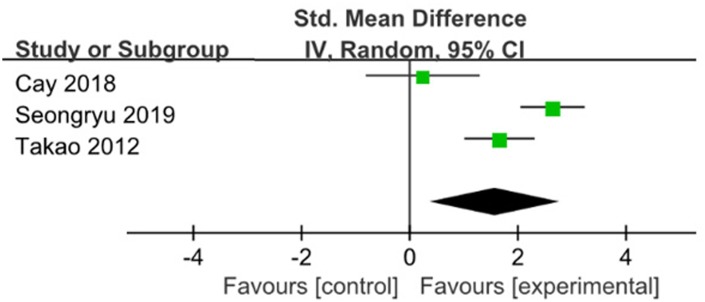
**The assessment of the effects of multicomponent exercise on attention by meta-analysis.**

Three studies used Digit Test were pooled with a significant effect in favor of ME for improving attention in 6 months [19, 20, 23] (SMD=1.58; 95%CI: 0.38 to 2.79, I2=88%) (Figure 4). Sensitivity analyses were done to explore potential sources of heterogeneity by excluding big sample size study, but the heterogeneity is relatively stable(SMD=2.16; 95%CI: 1.2 to 3.12; I2=79%), more high grade studies should be considered in future analysis.

### Executive function

**Figure 5:**
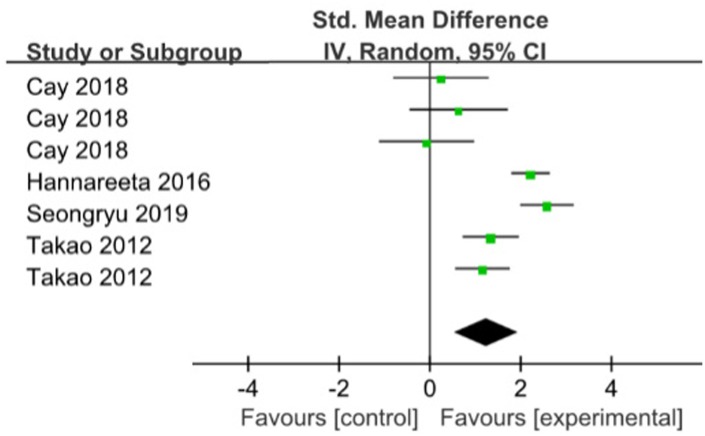
**The assessment of the effects of multicomponent exercise on executive function by meta-analysis.** Change in Total Cornell scores from baseline.

Executive Function Pooled analysis of four RCT indicated that those with ME had better executive function compared with those without (SMD=1.25; 95%CI: 0.57 to 1.93; I2=85%) [16, 19, 20, 23]. (Figure 5). When the trial with the big sample size was removed in a sensitivity analysis [19], compared with the control group, meta-analysis showed that ME had significance for improving executive function (SMD=0.80; 95%CI:0.28 to 1.31), with heterogeneity of 48% (p=0.10).

### Depression

Two studies reported data on depression outcomes for multicomponent intervention were included in the meta- analysis. One study indicated that there was no significant improvement (p=.438) for multicomponent intervention in reducing depression within the follow-up of 6 months completing treatment [18]. In another study, there are still more chances of both improvement and deterioration in the symptoms of depression among intervention group [20].

### BDNF

The review of BDNF included 3 studies. One study showed significant increase in BDNF levels compared to control group (p<0.05) [17]. The BDNF level in experimental group was significantly correlated with exercise dose based on average number of rides(r=0.50, p=.04) and related to improvements in ADAS (Alzheimer’s Disease Assessment Scale) (r=1.07, p=.01, independent of age, sex, educational level, and intervention) [22, 23].

## DISCUSSION

In order to avoid unnecessary disruptions to routine life and stress on families and resources, it is considerable to ameliorate physical and mental quality in old people with cognitive impairment.

Previous research on community exercise programs performed in the elderly usually excluded older adults with cognitive disorders and most duration are less than 6 months. Old community-dwellers can prevent or at least slow the hypofunction process of the ability to perform daily living through physical activity [24]. One meta-analysis showed beneficial effects on functional daily activities and balance from long-term home or community-based exercise programs for older people with cognitive impairment [25]. Lautens [26] reported that physical activity and behavioral interventions improve general cognitive function. A meta-analysis suggested that for patients with AD or MCI, physical exercise and cognitive training is the optimum intervention of cognitive and neuropsychiatric symptoms, respectively. Multicomponent exercise with moderate efficacy is the most safe intervention relatively. Additionally, the results suggest that non-pharmacological therapies are better than pharmacological therapies [27]. Heterogeneity was found significantly on global cognitive function outcomes and was significantly reduced when we used a subgroup analysis and conducted sensitivity analysis by omitting each trial one by one. This review supports a cost-effective, non-invasive method to improve cognition as an replacement to hospitalisation of high risk group.

Memory and attention deficits are among the earliest clinical manifestations of dementia [28]. In a recent randomized controlled trial [29], participants walked together in groups to finish aerobic exercises. The results would have shown increased memory and attention among those elderly adults in the aerobic exercise group if high attendance rate was taken in to consideration, only to a limited extent confirming the effectiveness of group based aerobic exercise in elder adults with MCI. However, we conclude that there is no significant improvement of memory in the multicomponent exercise group. Single or aerobic or non-aerobic physical exercise or physical exercise combined with an enriched environment benefits on attention tests in people with dementia [30, 31]. Also, combination benefits the brain greater than either alone, to some extent, it’s possible to suspect muliticomponent intervention are more positive. It might be that physical exercise stimulation may increase temporary arousal, or the other intervention components like group and pleasant activities could benefit concentration. After sensitivity analysis on memory outcomes by omitting each trial one by one, heterogeneity was reduced. The main issues of Han [16] and Danie [21]s' review are related to the established eligibility criteria and methodological evaluation, which maybe the reason of the questionable conclusion on memory. More high quality RCTs are needed to draw definite conclusions.

Physical exercise affected more on the functioning of the prefrontal cortex responsible for executive function than hippocampus responsible for memory in people with cognitive impairment [32]. In this study, we were focused on the impact of community-based multicomponent intervention on executive function, comparable studies for community-living people with moderate cognitive impairment are scarce. In a recent report, elderly adults with MCI performed aerobic exercise four times per week during 6 months meaningfully increased executive function (heart rate reserve was 75% to 85%) [33]. Reviews in older people in hospital with mild dementia showed that physical activity might have an effect on executive function instead of in community-dwellings [30, 34]. This meta-analysis shows that significant difference were observed in executive function between the groups, which is consistent with previous findings [35, 36] that the effect of single physical exercise or multicomponent interventions on executive function in older people with or without cognitive impairment.

Cognitive impairment is a risk factor for depression of older community-dwellers, in this review, there is no significant improvement for multicomponent intervention in reducing depression, high quality studies with larger sample sizes are required to test the efficacy of specific interventions. Conversely, a recent review established that exercise training reduces depression levels in people with dementia, however, the effect of exercise on depression was small and its clinical relevance is unclear [37]. Additionally, the identified psycho-social interventions are effective at reducing symptoms of depression or anxiety in people with dementia or mild cognitive impairment showed in a meta-analysis [38].

BDNF levels of active individuals are higher than those with inactive lifestyle, systemic BDNF increased by exercise would be expected to have positive effects on neuroplasticity and neuron survival and consequently improve cognitive performance [39, 40]. In this present review, one study showed significant increase of BDNF levels in multicomponent group compared to control group [17], consistent with one systematic review, physical exercise is positive on improving BDNF level, but it included participants without specific MCI criteria, acute effect of exercise and non RTC trials making it difficult to compare results [41]. One meta-analysis indicated that physical exercise can be a therapeutic choice up-regulating BDNF for MCI and AD patients, however, in this article, three studies showed high risk of attrition bias and two showed high risk of reporting bias [42].

Those conclusions should be interpreted bearing in mind other evidences that indicates positive effects of multicomponent exercise on cognition, attention and executive function for both individuals with MCI or AD. Even though there is an increasing number of researches concerning the effects and mechanisms of exercise over dysfunction in elderly, we have to explore that whether group-based multicomponent exercise influence these old patients. Older adults with cognitive impairment participate in community-based exercise programs might be a potential direction for future research as well as to coordinate health care patterns to accommodate social aging, we need further studies with therapist and carer availability and longer follow-up after training.

The fact that made comparing outcomes challenging we should consider is attention and executive function were not measured in an identical method in the eligible studies, and most studies measuring mood outcomes used a single test like a self-reported questionnaire. Additionally, this review only included trials published in English due to limited translation resources. Another limitation is the limited data available about the effect of group-based multicomponent exercise on BDNF outcomes and other neurotrophic biomarkers. Last, the total number of studies involved in the meta-analysis was too small to make subgroups analysis and only identify differences between the effects of frequency or duration of multicomponent exercise on cognition. This review included evidence from randomized, controlled trials and was reported in terms of PRISMA guidelines. This is the only one review to investigate, for community-dwelling adults with cognitive impairment, whether group-based multicomponent exercise influence cognition, memory, attention, executive function, depression and the level of BDNF.

In summary, our meta-analyses showed positive and beneficial effects of community-based multicomponent exercise on cognition, attention and executive function in community-dwelling older adults with mild cognitive impairment or Alzheimer's disease, but not on memory. However, there was limited data of the effect of multicomponent exercise on depression and biological markers for this group of people. Further research is warranted into the effects of community-based exercise programs for older adults with MCI or AD.

## MATERIALS AND METHODS

### Search strategy

The current systematic review and meta-analysis was performed according to the reported guidelines and was restricted to published studies that investigated the exercise effects in patients with MCI or dementia. The Pubmed, Embase and Medline were searched by one independent reviewer up to October 2019, without restrictions on nations. The search strategy used, individually or combined, the following subject words and their respective English synonyms: Exercise, Exercise Therapy, Exercise Movement Techniques, Multicomponent exercises, Aged, Dementia, Mild cognitive impairment, brain derived neurotrophic factor, nerve growth factor. Reference lists of the included trials and in reviews of the literature were used as additional source. The complete search strategy including PubMed, Embase and Medline is available upon request.

### Selection criteria and outcomes

Two reviewer performed a database search, study selection and data extraction. Titles and abstracts of papers were reviewed according to a standard screening checklist based on eligibility criteria. Full-text versions of selected RCT (potentially eligible and uncertain) were retrieved for complete review to determine eligibility. The third reviewer got the ultimate decision when disagreement persisted among reviewers. Measures of cognition, memory, attention, executive function and BDNF levels were the primary outcomes of this review.

### Data extraction and statistical analysis

Two reviewers extracted all data recorded as authors, publication year, number of cases, mean age of participants, study setting and outcomes. Disagreements between them were settled in consensus-based manner. Review Manager software was used to conduct post-intervention data in meta-analyses. A random-effects model was used for all meta analyses. For continuous data, mean differences or standardized mean difference were calculated with 95% CIs. When outcomes were assessed at multiple follow-up time points, data from the last exercise time point were chosen to be included in meta-analyses. Data homogeneity was assessed using the I^2^ statistic, where a value of > 50% indicated substantial heterogeneity. If substantial heterogeneity existed, included studies were significantly different to each other in terms of clinical factors, such as participant characteristics or intervention.
